# Large Transcalvarial Meningioma: Surgical Resection Aided by Preoperative Embolization

**DOI:** 10.7759/cureus.1229

**Published:** 2017-05-08

**Authors:** Ali S Haider, Haris Rana, Lyndon K Lee, Mrigank S Shail, Dean Leonard, Umair Khan, Richa Thakur, Tijani Osumah, Sam Finn, Kennith F Layton

**Affiliations:** 1 Texas A&M College of Medicine; 2 School of Medicine, Ross University; 3 School of Medicine, Xavier University School of Medicine; 4 School of Medicine, St. Georges University; 5 HSS Management; 6 Department of Radiology, Baylor University Medical Center

**Keywords:** meningioma, embolization, microsurgery, preoperative, hemorrhage, endovascular neurosurgery

## Abstract

Meningiomas are the most common type of primary brain tumors, accounting for about 30% of all brain tumors. Meningiomas originate from the meninges and can be associated with any part of the skull. Classification of meningiomas is based upon the World Health Organization (WHO) classification system and prognosis of meningiomas can be determined via histologic grading. Surgery is the gold standard treatment option for all types of meningiomas. Due to the high vascularity of some meningiomas, surgical resection can lead to certain complications including intraoperative blood loss and hemorrhage. Strategies for complication avoidance include preoperative embolization of the meningioma vascular supply. Preoperative embolization has been shown to assist in surgical resection of selected tumors and decrease intraoperative blood loss. We present a case of successful preoperative embolization for a large, complex, transcalvarial meningioma along with a literature review on this topic.

## Introduction

Meningiomas are the most common central nervous system neoplasms and can be highly vascular. Due to its increased vascularization of higher-grade tumors, preoperative embolization has been shown to assist in surgical resection of selected tumors. Although embolization has not been shown to decrease operative duration, complications, or degree of resection, it has been reported to decrease intraoperative blood loss and stabilize tumor growth. To determine if embolization is a reasonable approach, conventional angiography must be used to identify the vascular bed of the lesion and the feeding arterial branches [1]. We present a case of successful preoperative embolization for a large, complex, transcalvarial meningioma and review the literature on this topic.

## Case presentation

**Figure 1:**
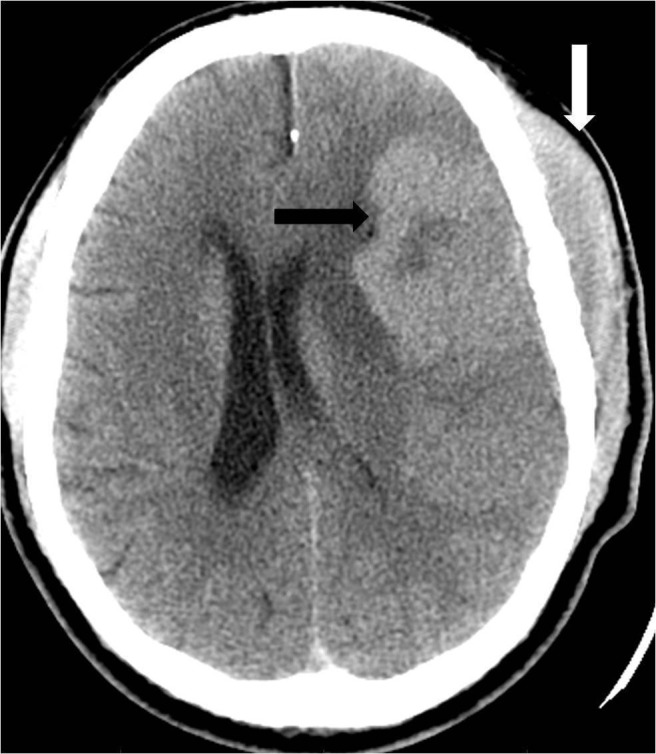
Noncontrast head computed tomography (CT) demonstrates a transcalvarial hyperdense mass with both intracranial (black arrow) and extracranial (white arrow) components.

**Figure 2:**
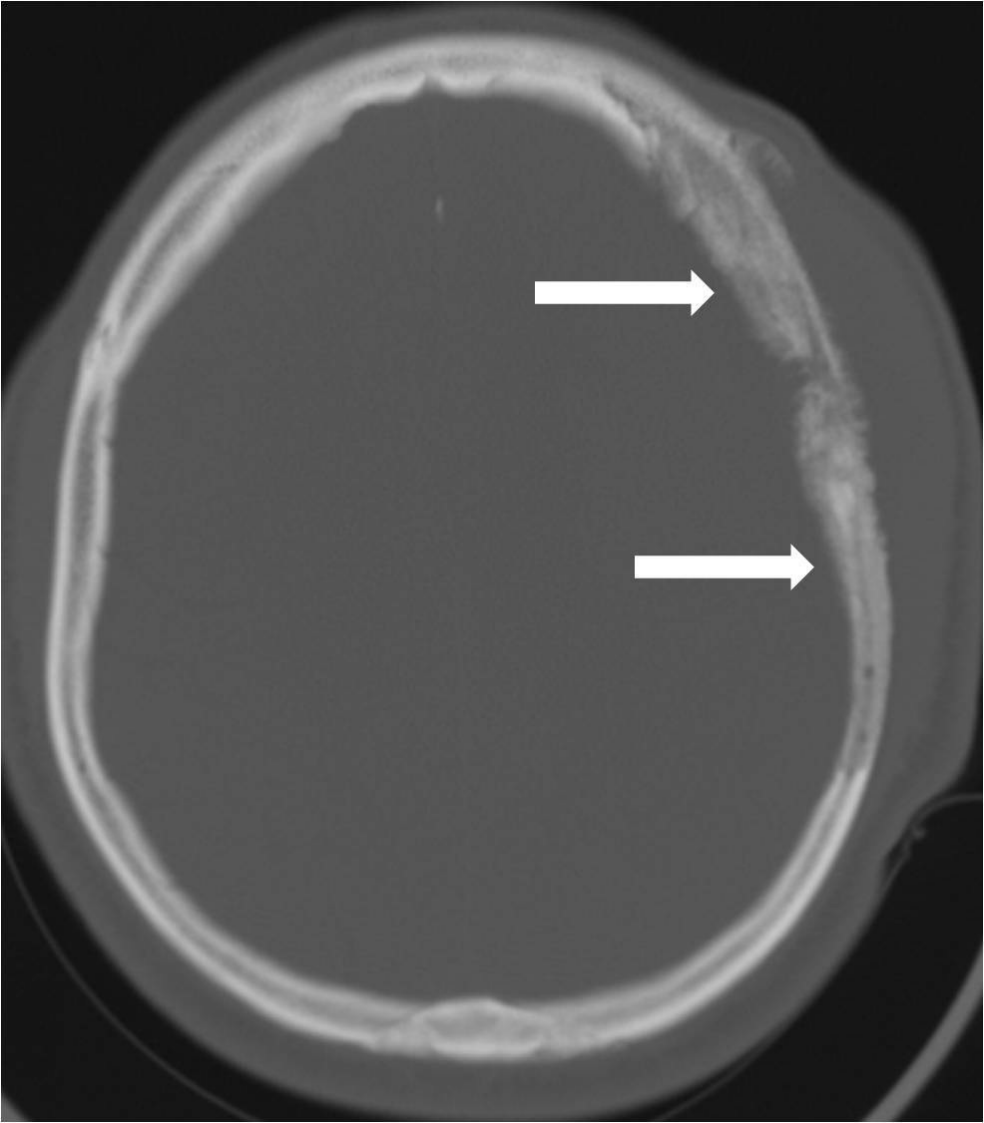
Bone windows from the head computed tomography (CT) reveal the osseous involvement with both osteolysis and hyperostosis of the left frontal and parietal calvaria (arrows).

**Figure 3:**
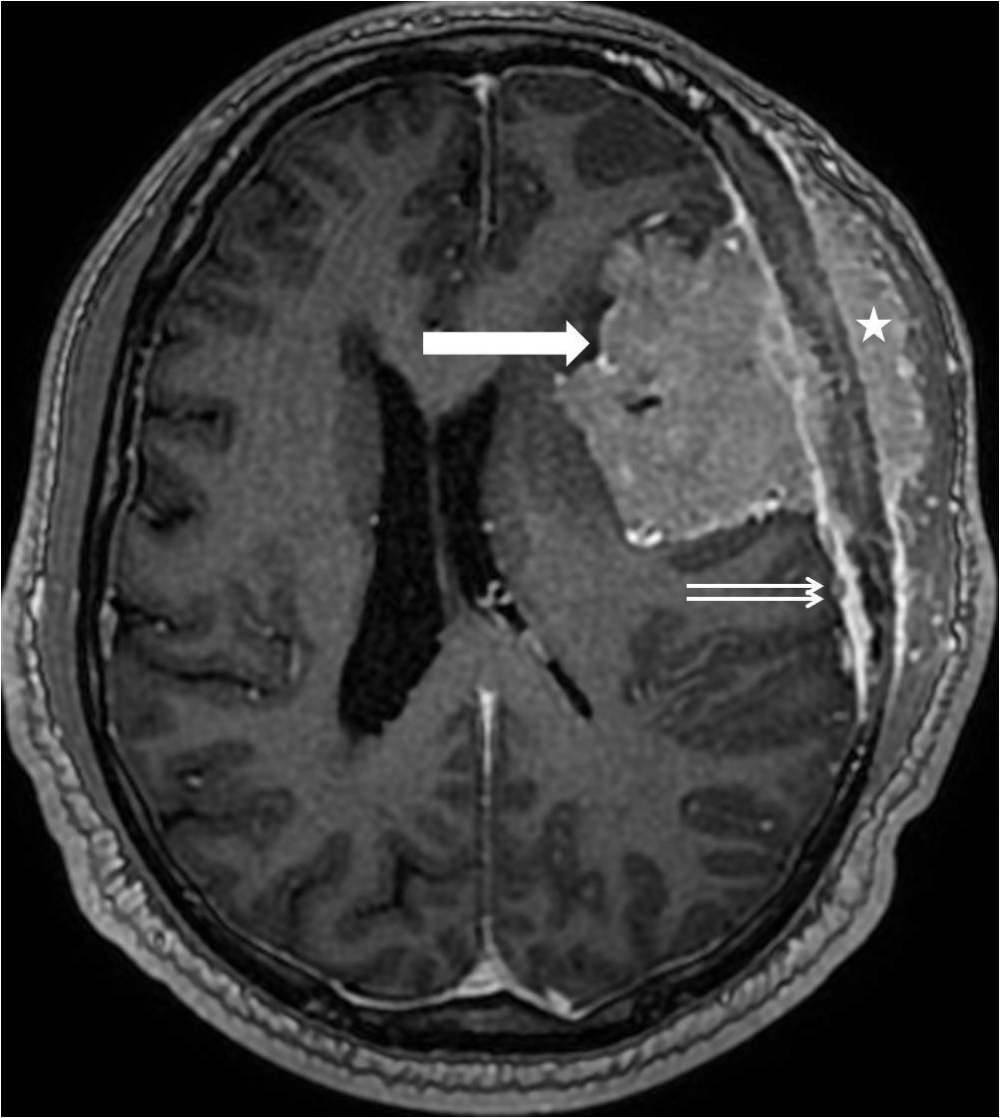
Axial T1 sequence after gadolinium contrast demonstrates the large trancalvarial mass with both intracranial (single arrow) and extracranial (asterisk) components. There is also adjacent thickening and enhancement of the dura (double arrows).

**Figure 4:**
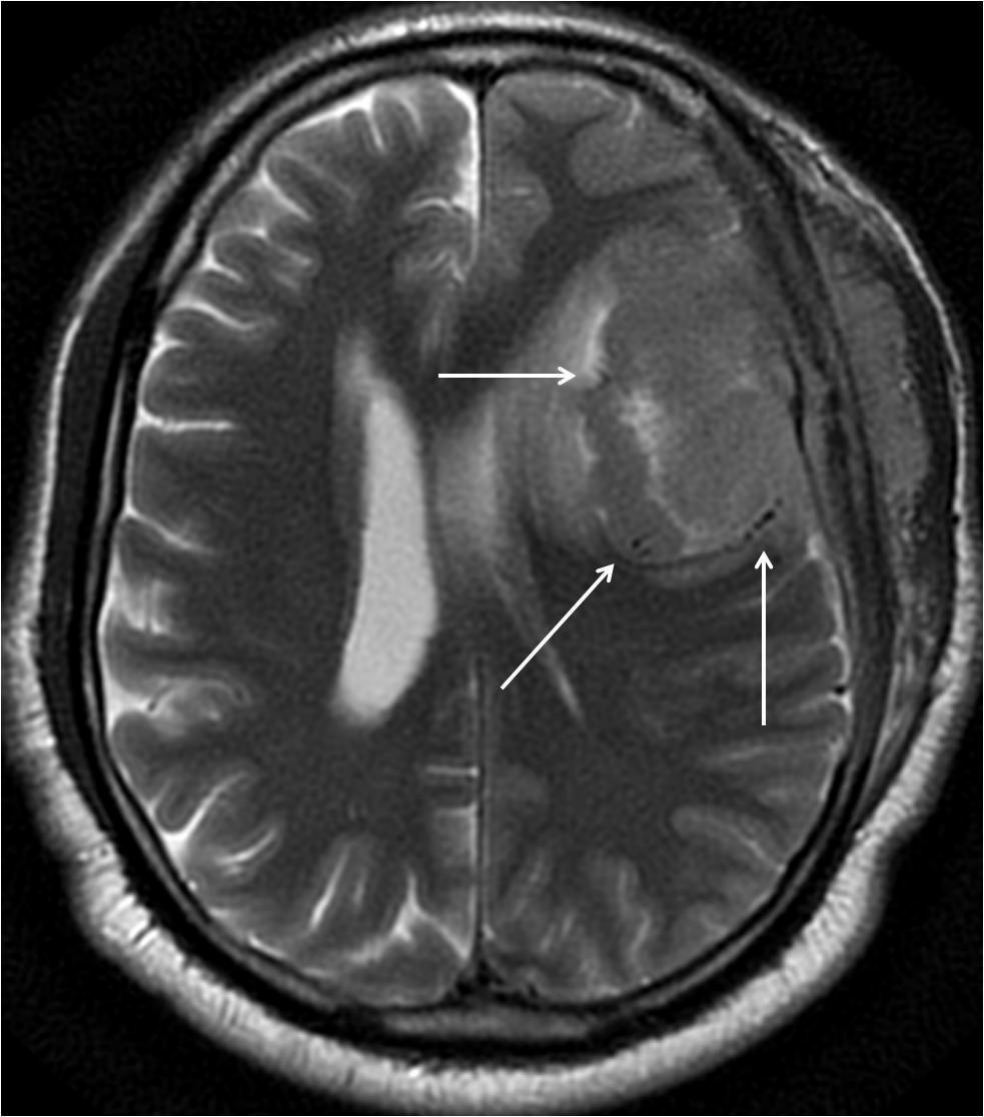
Axial T2 weighted magnetic resonance imaging (MRI) through the mass reveals multiple flow voids around the periphery of the tumor (arrows).

A 53-year-old African American male with a history of diabetes mellitus, hypertension, hyperlipidemia, and gastroesophageal reflux disease presented with complaints of headache, vision loss, brief twitching spells, and periodic unresponsiveness. Initial computed tomography (CT) imaging of the brain demonstrated a large hyperdense mass with intracranial and extracranial transcalvarial tumor extension (Figure 1). Bone windows from the CT study reveal the involvement of the calvaria (Figure 2). Subsequent brain magnetic resonance imaging (MRI) revealed an enhancing left frontal mass traversing the frontal and parietal bones, intracranial involvement of the left frontal lobe, and extracranial involvement of the suprazygomatic masticator space and scalp (Figure 3). The component deep to the inner table was associated with multiple peripheral flow voids (Figure 4). Vasogenic edema in the left frontal lobe was noted along with midline shift, effacement of the left lateral ventricle, and left uncal herniation. Following a seven-day dexamethasone preparatory course, the patient was re-admitted for preoperative embolization followed by surgery on the following day.

**Figure 5:**
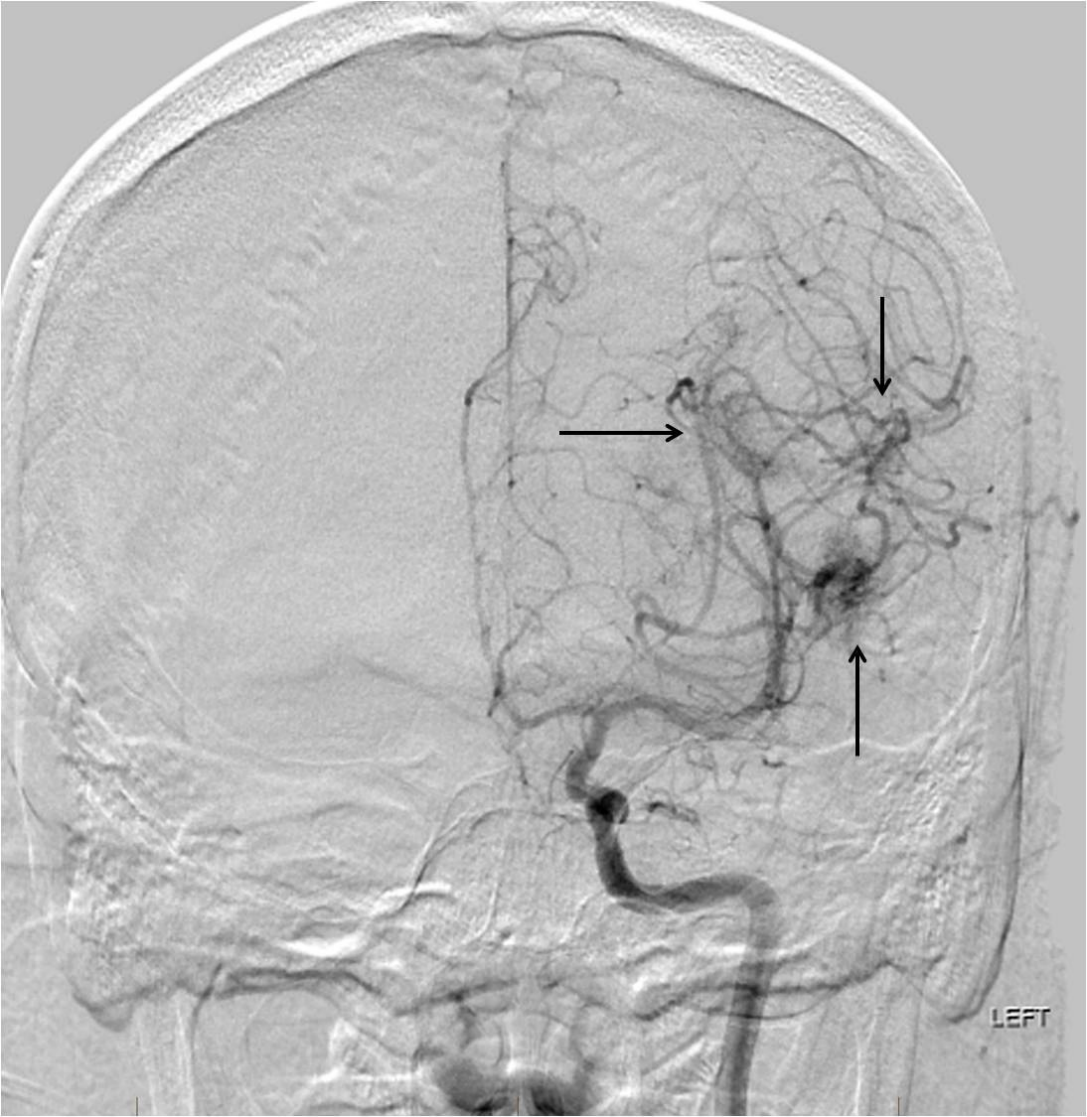
Left internal carotid artery digital subtraction angiography (DSA) reveals multifocal patchy intracranial enhancement related to the medial margin of the tumor and its interface with the underlying cerebral hemisphere (arrows). This portion of the tumor could not be safely devascularized preoperatively.

**Figure 6:**
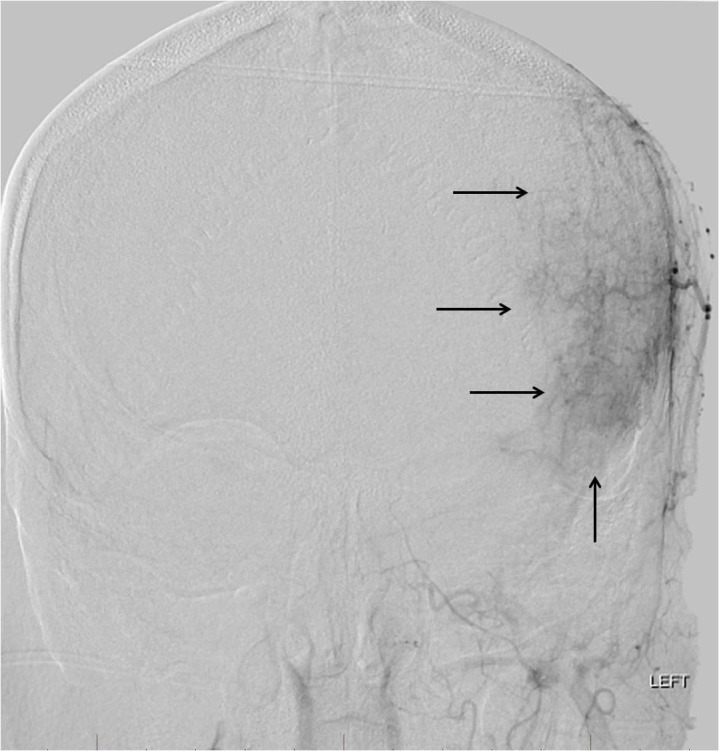
Left external carotid artery digital subtraction angiography (DSA) reveals a large area of tumor blush within the scalp and extending to involve the intracranial portion of the tumor (arrows). The tumor was supplied by numerous branches of the external carotid artery.

**Figure 7:**
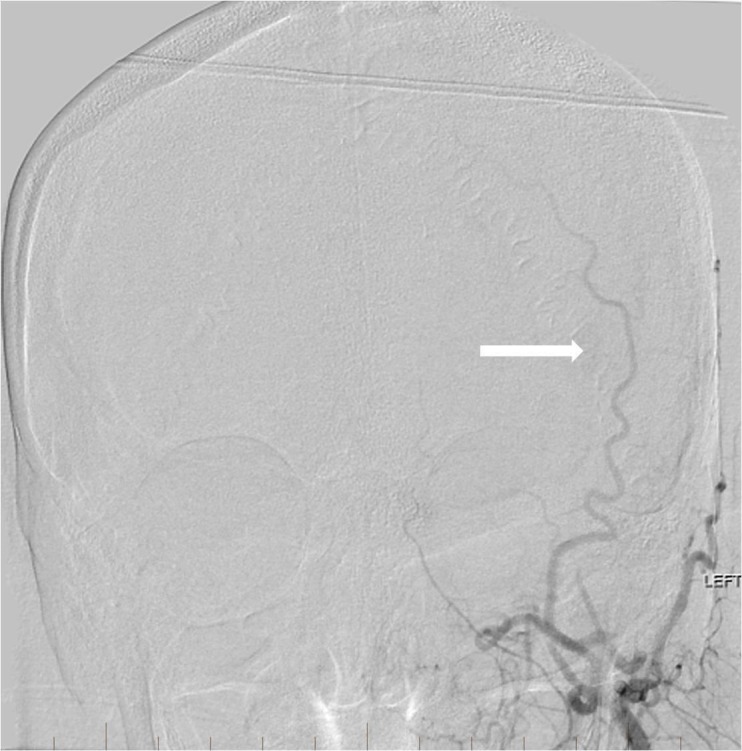
After endovascular embolization, repeat left external carotid artery digital subtraction angiography reveals no persistent tumor blush at the site of pretreatment abnormality (arrow). Only the normal appearing occipital and superficial temporal arteries are visible now.

Under conscious sedation, a 5 French sheath was placed in the right femoral artery, and a 5 French guiding catheter was advanced into the bilateral internal and external carotid arteries where digital subtraction angiography (DSA) was performed. DSA revealed vascular contributions to the tumor from the left middle cerebral, left middle meningeal, superficial temporal, anterior deep temporal, and middle deep temporal arteries (Figures 5-6). Arteriovenous shunting was present upon angiography of the left external carotid artery. Though the middle cerebral artery branches could not be safely embolized, endovascular embolization was performed in the left middle meningeal artery at the level of the foramen spinosum, left anterior deep temporal artery, left middle deep temporal artery, and the distal anterior and proximal portions of the left superficial temporal artery using a combination of polyvinyl alcohol (PVA) particles and gel foam torpedoes. Complete elimination of vascular supply from the left middle meningeal, left anterior deep temporal, and left middle deep temporal arteries along with near-complete elimination of contribution from the left superficial temporal artery was accomplished (Figure 7). The patient remained stable throughout and was scheduled for surgery the following day.

**Figure 8:**
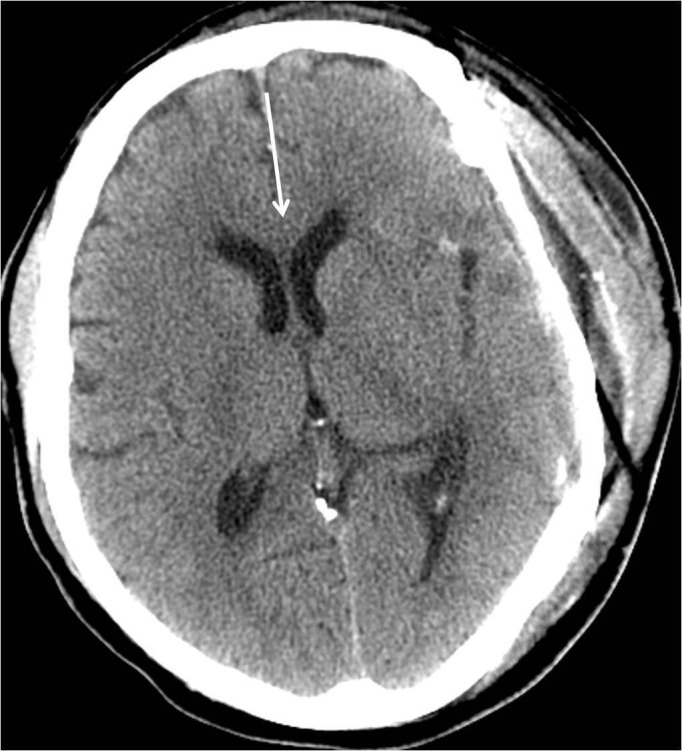
Postoperative head computed tomography (CT) prior to discharge reveals a left sided craniectomy and cranioplasty procedure with resection of the tumor components both intracranial and extracranial. There has been resolution of mass effect and midline shift (arrow).

Craniotomy was performed under general anesthesia and visual inspection showed tumor involvement of the temporalis muscle. Frozen sections of the tumor confirmed the diagnosis of meningioma. The tumor limits were outlined with the use of intraoperative navigation, and a burr hole was drilled away from the tumor margin in the parietal region. A craniectomy was performed into the temporal fossa, and the dura was opened and removed circumferentially. Microdissection was required to detach the middle cerebral artery from the tumor, and the tumor was subsequently circumscribed as vascular attachments were coagulated and divided. The middle cerebral artery branches were identified and preserved, and the tumor was removed with gross total resection followed by acrylic cranioplasty. The patient was returned to neuro-intensive care unit and experienced a postoperative seizure the day after the operation. Levetiracetam and lacosamide therapy was unable to adequately control the seizures and fosphenytoin was added, though multiple electroencephalograms (EEGs) were unable to identify clinical seizures or epileptiform changes. A postoperative CT of the head prior to discharge showed resolution of midline shift and resection of the hyperdense tumor with a small volume of expected postoperative blood products in the operative bed and stable frontal lobe vasogenic edema (Figure 8). The pathology report confirmed the tumor to be a World Health Organization (WHO) Grade II meningioma (atypical meningioma). The patient was later discharged on levetiracetam and fosphenytoin therapy as well as a dexamethasone taper, and upon discharge, the patient’s National Institutes of Health Stroke Scale (NIHSS) score was 0.

## Discussion

Meningiomas account for about 30% of all brain tumors. Meningiomas originate from the meninges and can be associated with any part of the skull [2]. Meningiomas are commonly located in the parasagittal area, the falx, cavernous sinus, tuberculum sellae, lamina cribrosa, foramen magnum, and torcular zones [3]. Meningioma classification is based upon the WHO classification system as shown: benign (Grade I) 90%, present as meningothelial, fibrous, transitional, psammomatous, angioblastic. Atypical (Grade II), seven percent, present as chordoid, clear cell, atypical. Anaplastic/malignant (Grade III), two percent, present as papillary, rhabdoid, anaplastic [4]. Histologic grading of meningiomas can be used for prognostic and therapeutic purposes. Generally, meningiomas are highly vascular tumors that obtain their blood supply from the arteries of the adjoining dura and bone although large and invasive tumors can recruit blood supply from cerebral and cerebellar arteries. Preoperative embolization of meningiomas is a procedure that is now frequently performed at centers with neurointerventional capability that can selectively embolize the target artery without harming healthy tissue. The greatest amount of meningioma softening is achieved around one week after embolization, causing greater softening of the tumor and relative ease of resection, less edema, and less blood loss [5]. In order to maximize safety and effectiveness, these procedures should only be performed at high volume neurosurgical centers with close collaboration between neurosurgeons and fellowship trained interventional neuroradiologists.

## Conclusions

Preoperative embolization is a proven ancillary management technique for intracranial meningiomas. It is most useful to the neurosurgeon when vascular supply from the skull base is surgically inaccessible such that initial devascularization in the initial stages of surgery is not feasible. The advantages of embolization include devascularization of the tumor leading to decreased operative blood loss, increased visualization of the operative field, and better resection of the tumor.
